# Whipple Grossing in the Era of New Staging: Should We Standardize?

**DOI:** 10.3390/diagnostics9040132

**Published:** 2019-09-29

**Authors:** Jiaqi Shi, Olca Basturk

**Affiliations:** 1 Department of Pathology, University of Michigan, Ann Arbor, MI 48109, USA; 2 Department of Pathology, Memorial Sloan Kettering Cancer Center, New York, NY 10065, USA; BasturkO@mskcc.org

**Keywords:** Whipple procedure, grossing, clinical stage, margin, pancreatic cancer

## Abstract

Whipple procedure, also known as pancreatoduodenectomy, is the most common surgery for the removal of tumors of the head of the pancreas, ampulla, distal common bile duct, or periampullary duodenum. It is also one of the most challenging resection specimens grossed by surgical pathologists. A thorough and consistent evaluation of the gross surgical specimen is the most critical first step for accurate diagnosis, determination of tumor origin, staging, and evaluation of margin status. However, there has been no standard grossing protocol for Whipple specimens, which has led to inaccurate diagnoses, staging, and inconsistent reporting. This issue has become even more challenging in the era of the size-based tumor staging systems recommended by the new 8th Edition of the American Joint Committee on Cancer (AJCC) Cancer Staging Manual. Moreover, new concerns have been raised regarding how to best evaluate margin status and lymph nodes. Studies have shown that different Whipple grossing methods can significantly impact margin assessment and lymph node yield and thus affect R0/R1 status and clinical stage. Other important issues under debate include nomenclature, definitions of margin (versus surface), and R1 status. Consistent Whipple grossing and standardization of reporting will provide better communication and more accurate diagnosis and staging, as well as prognostic prediction.

## 1. Introduction

The most common surgery to remove tumors of the head of the pancreas, ampulla, distal common bile duct, or periampullary duodenum is the Whipple procedure, which is also called pancreatoduodenectomy. For accurate diagnosis, evaluation of tumor origin, margin status, determining staging, and other important prognostic factors such as perineural invasion and lymphovascular invasion, the most critical first step in pathological evaluation is to correctly gross the surgical specimen and submit appropriate tissue sections for histologic assessment. Inadequate and/or inappropriate submission of tissue sections will lead to incorrect tumor stage, margin status, and even diagnosis (ampullary adenocarcinoma vs pancreatic ductal adenocarcinoma [PDA]) [1]. For example, under-sampling of pancreatic mucinous cystic neoplasms could have the potential risk of missing an associated small invasive adenocarcinoma component, therefore leading to misdiagnosis. Inappropriate sectioning and sampling of PDA could lead to inaccurate measuring of tumor size, which is currently the major factor used to determine the T stage in PDA. Incomplete submission of the retroperitoneal margin could lead to false R0 status. Additionally, proper classification and staging of ampullary cancers may not be possible because of certain approaches to sectioning the pancreatic head [2,3]. Therefore, the importance of grossing in pathologic evaluation of a Whipple specimen, and any surgical specimen, cannot be over-emphasized. However, due to the anatomic complexity and the relative rarity of the Whipple specimen, it is one of the most challenging resection specimens grossed by surgical pathologists. Even more importantly, the grossing protocol for Whipple specimens is not standardized, providing more room for mistakes and confusion.

Recently, the new 8th Edition of the American Joint Committee on Cancer (AJCC) Cancer Staging Manual has changed the way that PDA is staged [4,5]. Tumor size has become the only criterion for the T stage except for stage T4, and the N stage has been subdivided into N1 (1–3 lymph node metastasis) and N2 (≥4 lymph node metastasis). In addition, a tumor within 1 mm of margin is now considered to be a microscopic positive margin (R1). The new staging system was tested and was deemed to be more reproducible than the 7th edition of the AJCC by multiple studies including the original study analyzing the surveillance, epidemiology, and end results database [6], as well as by validation studies with a large multi-institutional cohort [5,7,8]. Even though one study did question the clinical relevance of subdividing the N stage [9], others reported better prognostic stratification with lymph node substaging [10,11]. The new R1 status based on the 1 mm rule of margin, but not the old R1 (tumor at the margin), was also shown to be an independent predictor for poor disease-free survival [12,13,14]. Given these significant changes, it may be time to reevaluate our current grossing protocols. Multiple studies have shown that different Whipple grossing methods can significantly impact margin assessment and lymph node count, and thus affect clinical stage and R0/R1 status [15,16,17]. Based on a meta-analysis, Demir et al. reported that lack of a standardized grossing protocol is one of the reasons why resection margin is not a valid prognostic marker in many studies [18].

## 2. Nomenclature Issue

When dealing with grossing and reporting of a Whipple specimen, the first item that needs to be standardized is the nomenclature. Multiple names have been assigned to the same anatomic region; moreover, the same name has been used to designate different compartments of the pancreatoduodenectomy. For example, “uncinate margin” was also known as “superior mesentery artery (SMA) margin”, “mesenteric margin”, or “retroperitoneal margin”. “Posterior margin” was used variably in different guideline texts referring to either the non-uncinate posterior free surface of the specimen, the uncinate margin itself, or the entire posterior region including the uncinate margin. Furthermore, there have also been debates about how to best name some of the “free surfaces” that come off readily without dissection; is it better that they be named “margins” or “free surfaces”? The inconsistency of nomenclature used by different institutions or countries could potentially lead to confusion during pathologic reporting and communications among physicians and large multi-institutional/international research studies. Therefore, a unified nomenclature is urgently needed to avoid confusion and standardize pathology reporting. 

## 3. Grossing Protocols and R0/R1 Status

Controversies exist as to how to standardize the Whipple grossing protocol and which protocol can provide the most accurate and necessary prognostic information. Recent studies using a standard grossing protocol have found that there is a significant difference in survival between R1 and R0 resection [18,19,20]. The two most commonly used grossing protocols are axial sectioning [15,21] and bivalving methods [2,17]. The axial sectioning method slices the specimen perpendicular to the longitudinal axis of the duodenum. This method requires detailed inking of all margins and complete submission of the pancreas, which is a very thorough method but can be cumbersome. More importantly, this method may lead to counting the same lymph node multiple times, and the recognition, proper classification, and staging of ampullary cancers may not be possible. The bivalving method requires probing of the main pancreatic duct and common bile duct, and the specimen is then sectioned along the plane defined by both probes. This method is beneficial, especially in evaluating intraductal lesions such as intraductal papillary mucinous neoplasms and ampullary tumors and does not require entire submission of the pancreas. In a recent comparative study, it was also found to be associated with a better lymph node yield and lymph node ratio [22]. However, R1 rate is reportedly lower in specimens grossed by the bivalving method, and inadequate sampling of the margins and free surfaces was thought to be the reason for the low R1 rate [15]. Because of the assessment of all margins and free surfaces, the axial sectioning method has been shown to yield a significantly higher R1 rate than the bivalving grossing method [23,24]. However, little is known about whether the higher R1 rate is better correlated with patient survival than the lower R1 rate determined by the bivalving method, and the definition of R1 is widely different in each study. Even though the axial sectioning method is more commonly adopted by European pathologists, and the bivalving method is more preferable in the US, which grossing protocol should be adapted is still up for debate.

Another question has also been raised because of the new “The margin is considered positive if the tumor is at or within 1 mm of the margin” rule for the uncinate margin in the 8th Edition of the AJCC Staging Manual. The manual does not mention if this rule also applies to other margins. Therefore, it is not clear if we need to change the margin evaluation method of other margins because the pancreatic neck and bile duct margins are currently submitted en face by many institutions in the US, and this method does not provide information on distance of tumor to the margins and therefore cannot determine if the tumor is within 1 mm of the margin. It is also not clear which margins/surfaces were included in each published study that led to the conclusion of the 1 mm rule for R1 status. If en face margins were not included in these studies, which presumably is the case since the distance of tumor to margin cannot be assessed in those margins, then keeping the current en face margin submission for certain margins may be appropriate. Furthermore, are representative margin sections sufficient for evaluation or do all margins need to be entirely submitted? The definition of tumors “at or within 1 mm of margin” also needs further clarification; does it refers to < 1 mm or ≤ 1 mm? Lastly, whether to consider tumors within 1 mm of the “free surface” as R1 needs consensus discussion as well.

## 4. Lymph Node Yield

There is evidence that adequate lymph node dissection and harvesting is critical for accurate staging of PDA and prognosis prediction; this is especially true with the recent substaging of nodal status in PDA [10]. Harvesting less than 12 lymph nodes from pancreatoduodenectomy in N0 patients has been associated with a worse patient outcome [25,26]. Therefore, the International Association of Pancreatology (IAP)/European Pancreatic Club consensus review of guidelines published in 2016 recommended that a minimum of 15 lymph nodes be examined histologically for correct staging [27]. It is known that different Whipple grossing protocols are associated with various lymph node yields [17,28]. Using a protocol known as the “Leeds” (axial sectioning) protocol proposed by Verbeke et al. [24,29], more lymph nodes were retrieved compared to the non-standardized protocol [28], although this method may lead to counting the same lymph node multiple times. A method of “orange-peeling”, where all peripancreatic soft tissues were removed before dissection, was also shown to yield a substantially higher number of lymph nodes [2,17]. Increased lymph node yield significantly increased lymph node-positive cases, which subsequently led to upstaging [17]. Even though a recent meta-analysis showed that there was no association between the total lymph nodes examined and overall survival in PDA in 12 out of 15 studies [30], positive lymph nodes and lymph node ratio are consistently associated with overall survival. Therefore, adequate lymph node dissection and evaluation is critical for accurate staging and prognosis.

## 5. Perspective

Standardization of the Whipple grossing protocol is essential for accurate pathologic staging and determination of important prognostic factors in PDA patients. In the era of the new AJCC staging manual, the Pancreatobiliary Pathology Society (PBPS) has formed a Grossing Working Group to evaluate the current practice patterns regarding Whipple specimen grossing and reporting, with the ultimate goal of establishing a standardized Whipple grossing protocol. The working group has conducted an international survey among pathologists, surgeons, and oncologists to obtain their views on critical issues regarding grossing and reporting of a Whipple specimen. We believe that the results, which will be published in the near future, will assist us in having a more global view of the current state and better understanding of the controversy related to Whipple grossing and reporting and will guide us towards our goal of standardizing the Whipple grossing and reporting protocol.

## Abbreviations

AJCC	American Joint Committee on Cancer
PDA	Pancreatic ductal adenocarcinoma
SMA	Superior mesentery artery
IAP	International Association of Pancreatology
PBPS	Pancreatobiliary Pathology Society
